# A Clinical Prediction Score for Intradialytic Hypotension Among Hospitalized Patients with Acute Kidney Injury

**DOI:** 10.3390/medsci14010080

**Published:** 2026-02-11

**Authors:** Piyanet Suwanin, Pattharawin Pattharanitima, Adis Tasanarong, Suthiya Anumas

**Affiliations:** 1 Division of Nephrology, Department of Internal Medicine, Faculty of Medicine, Thammasat University, Pathum Thani 12120, Thailand; piyanet.su@hotmail.com (P.S.); pattharawin@hotmail.com (P.P.); adis_tasanarong@hotmail.com (A.T.); 2 Chulabhorn International College of Medicine, Thammasat University, Pathum Thani 12120, Thailand

**Keywords:** hypotension, acute kidney injury (AKI), hemodialysis, risk assessment

## Abstract

**Background:** Intradialytic hypotension (IDH) in hospitalized patients with acute kidney injury (AKI) is associated with increased morbidity and mortality. Early identification of high-risk patients may enable preventive strategies. This study aimed to identify risk factors for IDH and develop a prediction model in this setting. **Method:** We conducted a retrospective cohort study of hospitalized patients with dialysis-requiring AKI who underwent conventional renal replacement therapy (RRT). Univariable and multivariable analyses were performed using generalized estimating equations (GEE) to account for repeated dialysis sessions within patients. IDH was defined as systolic blood pressure < 90 mmHg during dialysis. Although external validation was not performed, internal validation of the predictive model was conducted using 10-fold cross-validation. Model performance was assessed by calculating the area under the receiver operating characteristic curve (AUROC). **Result:** A total of 423 hemodialysis sessions from 85 patients were analyzed; the median age was 61 years, and the incidence of IDH session was 35.9%. Multivariable GEE analysis identified residual urine output <100 mL/day (OR 1.78, *p* = 0.007), vasopressor use (OR 3.36, *p* < 0.001), prior IDH (OR 2.25, *p* = 0.002), and lower pre-dialysis mean arterial pressure (MAP 80–89 mmHg: OR 2.43, *p* = 0.002; MAP < 80 mmHg: OR 2.95, *p* < 0.001) as significant predictors. Serum albumin < 2.5 g/dL was retained in the final model due to its clinical relevance and contribution to model performance despite borderline significance (OR 1.44, *p* = 0.08). A weighted integer-based risk score was derived directly from the coefficients of the final multivariable GEE model, stratifying patients into low-, intermediate-, and high-risk groups with IDH incidences of 11.6%, 33.9%, and 56.7%. The model demonstrated good discrimination, with an AUROC of 0.760 (95% CI, 0.714–0.807). **Conclusions:** The predictive score for IDH demonstrated good performance and highlights the importance of raising awareness to guide interventions aimed at improving the outcomes of hospitalized AKI patients requiring conventional RRT.

## 1. Introduction

Acute kidney injury (AKI) affects approximately 20–25% of hospitalized patients, of whom about 12% develop stage 3 AKI requiring dialysis [1,2]. This severe form of AKI is associated with poor clinical outcomes, including increased mortality, reduced quality of life, and progression to chronic kidney disease (CKD) or end-stage kidney disease (ESKD) [1,2,3]. Thus, AKI requiring renal replacement therapy affects not only short-term outcomes but also long-term outcomes, particularly in patients who do not achieve renal recovery [4].

Intradialytic Hypotension (IDH) is a common and severe complication of hemodialysis characterized by a sudden drop in blood pressure during or immediately following a treatment session. According to the KDOQI (Kidney Disease Outcomes Quality Initiative) and ERBP (European Renal Best Practice) guidelines, IDH is most frequently defined as decrease in systolic blood pressure (SBP) by ≥20 mmHg or a reduction in mean arterial pressure (MAP) by ≥10 mmHg, accompanied by symptoms such as abdominal discomfort, yawning, nausea, vomiting, muscle cramps, restlessness, dizziness, anxiety, and cardiovascular or neurological compromise [5,6]. The Japanese Society for Dialysis Therapy (JSDT) guidelines define IDH as a symptomatic sudden decrease in SBP of ≥30 mmHg or a decrease in MAP of ≥10 mmHg [7]. Recent evidence also emphasizes “nadir-based” definitions specifically an absolute nadir SBP < 90 mmHg as being the most strongly associated with increased cardiovascular mortality and poor patient outcomes [8].

IDH arises from an imbalance between ultrafiltration-induced plasma volume removal and the body’s compensatory capacity, including plasma refilling, vascular tone, and cardiovascular reserve. When fluid removal exceeds these compensatory mechanisms, effective circulating volume falls, resulting in hypotension and organ hypoperfusion [9].

IDH acts as a critical barrier to renal recovery and a potent driver of systemic morbidity. Because the injured kidney frequently loses the capacity for hemodynamic autoregulation, even transient drops in blood pressure during dialysis result in direct proportional reductions in renal blood flow, triggering repetitive “second-hit” ischemic injuries to the recovering tubular cells [10]. Beyond the kidneys, IDH-induced hypoperfusion extends to the myocardium (myocardial stunning), significantly escalating the risk of cardiovascular events and mortality [11]. Consequently, IDH is no longer viewed merely as a side effect of fluid removal, but as a primary determinant of whether a patient transitions from AKI to ESKD or survives the acute illness [12].

Several studies have identified risk factors associated with IDH, including cardiovascular disease, poor nutritional status, hypoalbuminemia, female sex, low pre-dialysis systolic blood pressure (<100 mmHg), and severe anemia [13]. Current evidence on prediction tools for IDH in patients with acute kidney injury undergoing renal replacement therapy has primarily focused on critically ill populations and often relies on invasive hemodynamic monitoring, laboratory-based parameters, or complex predictive models. For example, the SOCRATE score incorporates the cardiovascular SOFA score, capillary refill time, and lactate levels, while machine learning–based models require high-resolution physiological and laboratory data [14]. These requirements may limit the feasibility and generalizability of such tools in routine clinical practice and resource-limited settings.

Therefore, this study aimed to identify risk factors for intradialytic hypotension (IDH) in hospitalized patients with AKI receiving conventional renal replacement therapy. In addition, we sought to develop a practical, easy-to-use prediction model based on routinely available clinical variables, suitable for implementation in real-world and resource-limited settings, in a population that differs substantially from previously studied critical care cohorts.

## 2. Methods

### 2.1. Study Design and Participants

This study was a retrospective study conducted between 1 January 2024 and 31 December 2024 at Thammasat University Hospital, Pathum Thani, Thailand. Data were extracted from electronic medical records and a review of hemodialysis sheet. The inclusion criteria consisted of hospitalized patients who were older than 18 years old with diagnosed AKI and undergoing conventional intermittent hemodialysis treatment. AKI was defined based on the KDIGO clinical practice guidelines [15]. Patients with ESKD, those receiving alternative renal replacement modalities (including peritoneal dialysis or continuous renal replacement therapy), patients with permanent pacemakers, pregnant patients, kidney transplant recipients, and those receiving midodrine were excluded. In addition, patients requiring more than moderate doses of vasopressor agents and those with MAP < 65 mmHg were excluded to ensure that the study specifically evaluated predictors of dialysis-related hypotension rather than hypotension driven by underlying hemodynamic instability.

The primary objective of this study was to identify the risk factors associated with intradialytic hypotension events in hospitalized patients with diagnosed AKI. Based on definitions of IDH defined as a reduction in SBP below 90 mmHg. This nadir-based definition was selected based on evidence demonstrating that absolute nadir SBP is more strongly associated with mortality and adverse outcomes [8]. The data collected encompassed patient-specific factors, such as demographic information, underlying diseases, the cause of AKI, baseline creatinine levels, residual urine output, and pre-dialysis blood pressure. Disease-related factors, including vascular access, pre-dialysis laboratory results, and dialysis prescriptions, were also evaluated. The secondary objective was to develop and validate a predictive model for intradialytic hypotension in AKI patients.

### 2.2. Statistical Analysis

Statistical data were presented as mean with standard deviation (SD) or median with interquartile range, according to data distribution; categorical variables were presented as frequencies and percentages. To assess the differences in baseline characteristics between the two groups for categorical and continuous data, the Fisher exact test and Mann–Whitney test were used, respectively. Univariable and multivariable analyses were performed using generalized estimating equations (GEE) to identify risk factors for IDH while accounting for repeated dialysis sessions within the same patient. Variables with a *p* value < 0.10 in the univariable GEE analysis were entered into the multivariable GEE model. Odds ratios (ORs) with 95% confidence intervals (CIs) were reported to quantify the strength of associations, and a *p* value < 0.05 was considered statistically significant. To construct the prediction score, the regression coefficients (log odds) from the significant predictors and clinically relevant variables identified in the multivariable GEE model were used. Internal validation of the model was performed using 10-fold cross-validation. These log odds were then converted into integer score points by dividing them by the smallest absolute coefficient. The resulting values were rounded to generate practical score points for outcome prediction. Risk categories were defined empirically based on the distribution of total scores and the observed incidence of IDH across score levels in the study population. The performance evaluation of the prediction scores was accomplished by calculating the area under the receiver operating characteristic curve (AUROC). All statistical analysis was performed using Stata 17.0/BE.

### 2.3. Ethical Considerations

This study was conducted in accordance with the Declaration of Helsinki and approved by the Human Research Ethics Committee of Thammasat University (Medicine) under project number MTU-EC-IM-0-042/67, dated 30 May 2024.

## 3. Results

### 3.1. Baseline Characteristics

**Figure 1:**
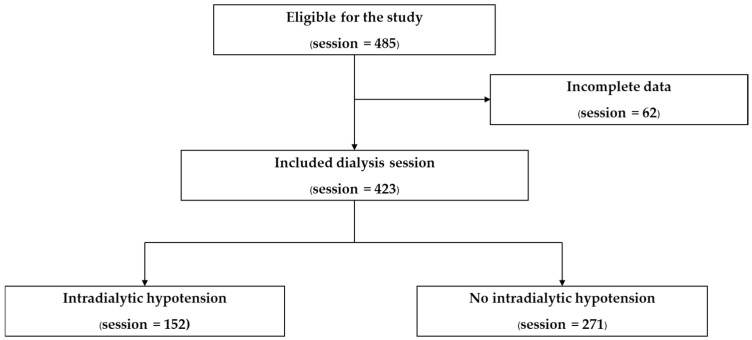
Flow diagram of dialysis session selection.

A total of 485 hemodialysis sessions from 91 patients were initially enrolled; of these, 423 sessions from 85 patients were included in the final analysis (Figure 1). The remaining 62 sessions were excluded due to incomplete data, primarily missing urine output records and pre-dialysis laboratory results required for analysis. Patient-level baseline characteristics showed that more than half of the cohort were male (58.8%), with a median age of 61 years (interquartile range [IQR], 47–73 years). The median baseline serum creatinine level was 1.3 mg/dL (IQR, 0.95–1.94 mg/dL). The most prevalent comorbidities were hypertension (61.2%), dyslipidemia (40.0%), diabetes mellitus (38.8%), and ischemic heart disease (23.5%). The etiologies of AKI were heterogeneous, with sepsis (22.4%) and cardiorenal syndrome (21.2%) being the most common causes (Table 1).

Session-level analysis demonstrated that IDH occurred in 35.9% of dialysis sessions and was most frequent among patients with residual urine output < 100 mL/day, accounting for 61.8% of all IDH episodes. In contrast, patients with residual urine output of 100–399 mL/day and ≥400 mL/day had significantly lower incidences of IDH, at 21.1% and 17.1%, respectively (*p* < 0.001). Patients receiving vasopressor therapy (limited to norepinephrine in this study) experienced IDH in 32.2% of sessions compared with 8.9% in sessions without IDH (*p* < 0.001). Similarly, a prior history of IDH was associated with a higher incidence of IDH (44.1% vs. 17.7%, *p* < 0.001). Pre-dialysis blood pressure parameters, including SBP, diastolic blood pressure (DBP), MAP, and serum sodium were significantly lower in sessions complicated by IDH. In contrast, pre-dialysis laboratory parameters including blood urea nitrogen (BUN), serum creatinine, electrolytes (except serum sodium), serum albumin, hematocrit, and blood glucose did not differ significantly between sessions with and without IDH. Notably, dialysis prescription variables, such as dialysate flow rate, blood flow rate, dialysate sodium, potassium, and calcium concentrations, prescribed ultrafiltration volume, and session duration, were also comparable between the two groups (Table 2).

### 3.2. Risk Factors of IDH in AKI

Univariable GEE analysis identified several factors associated with an increased risk of IDH among patients with AKI, including residual urine output < 100 mL/day, use of vasopressor agent, a history of IDH in previous dialysis session, lower pre-dialysis MAP, and serum albumin < 2.5 g/dL.

Variables with *p* < 0.10 in the univariable analysis were subsequently entered into the multivariable GEE model. In the adjusted analysis, residual urine output < 100 mL/day, vasopressor use, a prior history of IDH, and lower pre-dialysis MAP remained independently associated with an increased risk of IDH (Table 3).

### 3.3. The Prediction Scores

Variables independently associated with IDH in the multivariable GEE model including residual urine output < 100 mL/day, vasopressor use, lower pre-dialysis MAP, and a prior history of IDH were incorporated into the prediction model to develop a clinical risk score for patients with AKI. In addition, serum albumin < 2.5 g/dL was retained due to its clinical relevance and contribution to model performance despite borderline statistical significance (OR 1.44, *p* = 0.08). Internal validation was performed using 10-fold cross-validation to evaluate the discriminatory performance of the model. The cross-validated model demonstrated good discrimination, with an AUROC of 0.733 (95% confidence interval [CI], 0.617–0.914).

Regression coefficients from the final multivariable GEE model were used to construct the scoring system. The smallest coefficient among the retained predictors (β = 0.36) was selected as the reference value and used as the denominator to standardize the remaining coefficients (Table 4). These standardized coefficients were subsequently rounded to the nearest integer to generate a practical, integer-based risk score. The individual predictors and their corresponding point allocations are summarized in Table 5.

**Figure 2:**
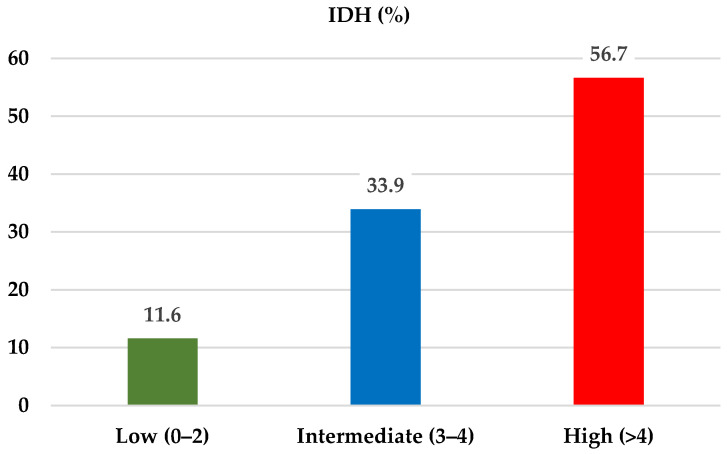
Incidence of intradialytic hypotension (IDH) according to risk score categories.

After applying the predictive model to our study population, patients with AKI were stratified into three risk groups based on the total risk score. Risk categories were defined empirically based on the distribution of total scores and the observed incidence of IDH across score levels in the study population. The prevalence of IDH was 11.6% in the low-risk group (score ≤ 2), 33.9% in the intermediate-risk group (score 3–4), and 58.9% in the high-risk group (score > 4) (Figure 2).

**Figure 3:**
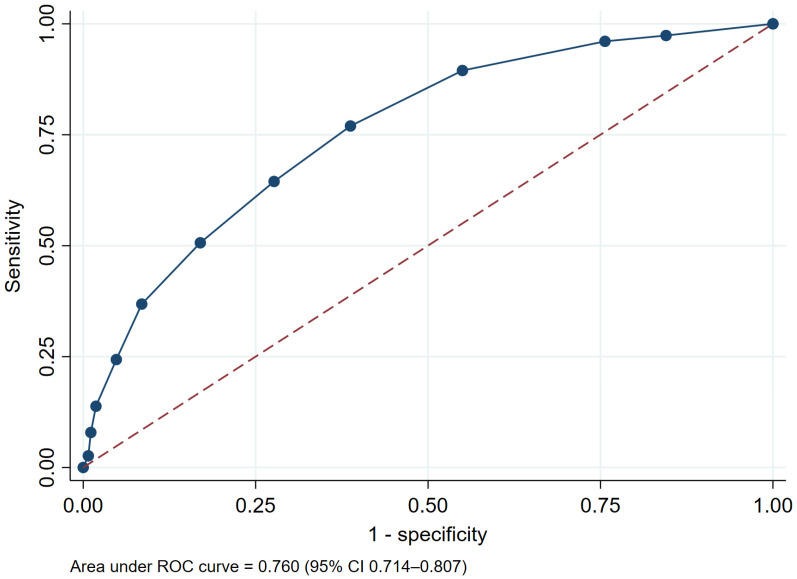
Receiver operating characteristic (ROC) curve of the prediction model for intradialytic hypotension (IDH). The blue solid line represents the performance of the prediction model, and the red dashed line represents the reference line of no discrimination (AUROC = 0.5).

The integer-based risk score also demonstrated good discriminative performance, with an AUROC of 0.760 (95% CI, 0.714–0.807) (Figure 3).

## 4. Discussion

This study identified several clinical factors independently associated with an increased risk of IDH in patients with AKI, including residual urine output < 100 mL/day, use of vasopressor agent, a history of IDH in previous dialysis session, and lower pre-dialysis MAP, particularly in the ranges of 80–89 mmHg and <80 mmHg. A predictive model incorporating these factors, along with serum albumin < 2.5 g/dL due to its clinical relevance and contribution to model performance, demonstrated good discriminative ability, with an AUROC of 0.760.

In our cohort, IDH was defined as a nadir SBP < 90 mmHg. This approach may capture events that are clinically silent yet hemodynamically significant, while potentially excluding symptomatic episodes that do not reach this threshold. However, evidence suggests that nadir SBP is more strongly associated with adverse outcomes [8], supporting the clinical relevance of this definition. Therefore, while this approach enhances objectivity and reproducibility in retrospective data, caution is warranted when directly comparing our results with studies employing alternative IDH definitions.

Findings from the session-level baseline comparison and the GEE model demonstrated that IDH was primarily associated with patient-related hemodynamic factors, including lower pre-dialysis blood pressure, reduced residual urine output, vasopressor use, and a prior history of IDH. In contrast, dialysis prescription parameters, including dialysate composition and UF prescriptions, did not differ significantly between IDH and non-IDH sessions and were not independently associated with IDH.

Dialysis prescription variables are traditionally considered important contributors to IDH through several physiological mechanisms. High UF rates may exceed the capacity for plasma refilling from the interstitial space, leading to a decline in effective circulating volume. In addition, dialysate sodium, calcium concentration, and temperature influence vascular tone, autonomic responses, and myocardial contractility, which are critical determinants of hemodynamic stability during dialysis [16,17]. However, this expected association was not observed in our cohort. This likely reflects the relatively standardized and conservative dialysis practices applied in hospitalized patients with AKI, where UF targets and dialysate settings are carefully adjusted to minimize hemodynamic instability. Consequently, limited variability in dialysis prescriptions may have reduced their observable impact on IDH risk and explains why these parameters were not included in the final model. In this context, patient-related hemodynamic factors appeared to play a more dominant role than modifiable dialysis settings in the development of IDH.

A residual urine output of <100 mL/day was significantly associated with an increased risk of IDH in our cohort. This finding suggests that, despite similar prescribed ultrafiltration rates, patients with lower residual urine output may have had a larger cumulative fluid burden, potentially related to greater interdialytic weight gain, leading to inadequate cardiovascular compensation during ultrafiltration and increased susceptibility to IDH [18]. However, interdialytic weight gain was not measured in this study, and this proposed mechanism could not be directly confirmed. Additionally, severe loss of RRF may be associated with accumulation of uremic toxins not adequately reflected by BUN levels. These toxins may impair vascular tone, endothelial function, and myocardial contractility [19,20], thereby further reducing the ability to compensate for intradialytic volume shifts and increasing the risk of IDH.

We also observed that vasopressor use and lower pre-dialysis MAP were independently associated with an increased risk of IDH, despite the exclusion of patients receiving moderate doses of vasopressors. These findings are consistent with prior studies [21,22], which have reported an association between vasoactive agent use and IDH in critically ill patients.

In the multivariable GEE model, serum albumin < 2.5 g/dL showed a borderline association with IDH but was retained in the prediction model due to its clinical relevance and contribution to model performance. Hypoalbuminemia may reflect poor nutritional status, systemic inflammation, and reduced plasma oncotic pressure, all of which can impair effective plasma refilling during ultrafiltration. Reduced oncotic pressure diminishes the gradient for fluid movement from the interstitial to the intravascular compartment, thereby predisposing patients to intravascular volume depletion during dialysis [16,23]. These mechanisms provide physiological plausibility for the observed association between low serum albumin and increased susceptibility to IDH, despite borderline statistical significance in the adjusted analysis.

Given the limited number of studies developing prediction scores for IDH in patients with AKI requiring renal replacement therapy, our study provides a predictive score with several notable strengths. These include the integration of simple, non-invasive variables into the scoring system, incorporating readily available patient characteristics and vasopressor use. This approach eliminates the need for data obtained from invasive procedures, thereby enhancing rapid applicability across diverse patient populations and facilitating implementation in routine clinical practice. In addition, the use of GEE allowed us to appropriately account for repeated dialysis sessions within the same patient, strengthening the robustness of the session-level analysis. Nevertheless, several limitations should be acknowledged. This was a single-center study, which may limit the generalizability of the findings. Although internal validation was performed, the predictive score has not yet undergone external validation. Finally, pre-dialysis body weight was unavailable, precluding estimation of interdialytic weight gain (IDWG).

Despite these limitations, incorporating this risk score into routine clinical practice may improve early risk stratification and promote timely preventive interventions in patients at high risk for IDH, such as the use of sodium profiling, dialysate cooling, and closer hemodynamic monitoring. These strategies may help reduce IDH episodes, which are known to adversely affect long-term kidney outcomes and patient survival. Further multicenter studies incorporating detailed volume-related parameters, including IDWG, and external validation cohorts are warranted to assess the generalizability and robustness of this predictive tool.

## 5. Conclusions

The predictive model for IDH in patients with AKI incorporated independent risk factors including urine output < 100 mL/day, vasopressor use, a prior IDH episode, and lower pre-dialysis mean arterial pressure (80–89 mmHg and <80 mmHg). Serum albumin < 2.5 g/dL was additionally included due to its clinical relevance and contribution to model performance, despite borderline statistical significance. The model demonstrated good discriminative performance. These findings may help clinicians recognize patients at higher risk of IDH and support individualized dialysis management. Further external validation in larger, multicenter populations may be required before routine clinical application.

## Figures and Tables

**Table 1:** Patient-level baseline characteristics of the study population.

Characteristic	*n* = 85
Age (year), median (IQR)	61 (47–73)
Male, *n* (%)	50 (58.8)
Body weight (kg), median (IQR)	60 (55–70)
Height (cm), median (IQR)	160 (155–169)
Underlying diseases, *n* (%) -Hypertension -Dyslipidemia -Type 2 diabetes -Ischemic heart disease -Cerebrovascular disease -Atrial fibrillation -Peripheral arterial disease	52 (61.2) 34 (40.0) 33 (38.8) 20 (23.5) 7 (8.2) 6 (7.1) 3 (3.5)
Cause of CKD, *n* (%) -None/unknown cause -Hypertension -Diabetic nephropathy -Glomerulonephritis	75 (85.9) 6 (7.1) 4 (4.7) 2 (2.4)
Cause of AKI, *n* (%) -Sepsis -Cardiorenal syndrome -Ischemic acute tubular necrosis -Non-ischemic acute tubular necrosis -Hypovolemia -Others	19 (22.4) 18 (21.2) 13 (15.3) 13 (15.3) 14 (16.5) 8 (9.4)
Baseline creatinine (mg/dL), median (IQR)	1.30 (0.95–1.94)

Abbreviation: AKI, acute kidney injury; CKD, chronic kidney disease; IQR, interquartile range.

**Table 2:** Session-level characteristics of hemodialysis sessions comparing intradialytic hypotension (IDH) and non-IDH events.

Characteristic	All (*n* = 423)	IDH (*n* = 152)	No IDH (*n* = 271)	*p*-Value
Urine output (mL/day), median (IQR) -≥400 (mL/day), *n* (%) -100–399 (mL/day), *n* (%) -<100 (mL/day), *n* (%)	105 (15–605) 141 (33.3) 79 (18.7) 203 (48.0)	48 (10–305) 32 (21.1) 26 (17.1) 94 (61.8)	220 (25–750) 109 (40.2) 53 (19.6) 109 (40.2)	<0.001 <0.001
-Receiving vasopressor drugs, *n* (%)	73 (17.3)	49 (32.2)	24 (8.9)	<0.001
-Previous IDH, *n* (%)	115 (27.2)	67 (44.1)	48 (17.7)	<0.001
Vascular access, *n* (%) -DLC at femoral vein -DLC at internal jugular vein	353 (83.5) 70 (16.6)	133 (87.5) 19 (12.5)	220 (81.2) 51 (18.8)	0.10
Dialyzer, *n* (%) -EL150 -EL170 -EL210	381 (90.1) 41 (9.7) 1 (0.2)	134 (88.7) 17 (11.2) 1 (0.7)	247 (91.1) 24 (8.9) 0 (0)	0.29
Pre-dialysis laboratory results, median (IQR) -BUN (mg/dL) -Creatinine (mg/dL) -Sodium (mEq/L) -Potassium (mEq/L) -Bicarbonate (mEq/L) -Calcium (mg/dL) -Phosphate (mg/dL) -Albumin (g/dL) -Hematocrit (%) -Glucose (mg/dL)	98 (71–122) 5.33 (3.99–7.32) 135 (132–139) 4.2 (3.7–4.7) 20 (18–23) 8.6 (8.1–9.0) 5.3 (4.1–6.8) 2.6 (2.3–3.0) 23.9 (21.4–26.7) 144 (122–168)	100 (74–209) 5.04 (4.00–7.04) 136 (133–140) 4.3 (3.7–4.8) 20 (18–22) 8.6 (8.1–9.1) 5.3 (4.1–6.5) 2.5 (2.3–2.9) 24.1 (21.7–26.9) 145 (123–178)	96 (70–121) 5.52 (3.98–7.41) 135 (132–138) 4.1 (3.7–4.7) 20 (18–23) 8.5 (8.1–8.9) 5.2 (4.1–7.1) 2.7 (2.4–3.0) 23.8 (21.2–26.6) 142 (122–165)	0.27 0.16 0.04 0.34 0.14 0.37 0.49 0.08 0.39 0.11
HD prescription, median (IQR) -Blood flow rate (mL/min) -Dialysate flow rate (mL/min) -Dialysate sodium (mEq/L) -Dialysate potassium (mEq/L) -Dialysate calcium (mEq/L) -Prescribed ultrafiltration volume (L) -Duration of HD (hours)	245 (200–300) 499 (500–500) 138 (138–138) 2 (2–3) 2.5 (2.5–3.5) 2.0 (1.2–2.8) 4 (4–4)	243 (200–250) 500 (500–500) 138 (138–138) 2 (2–3) 2.5 (2.5–3.5) 2.0 (1.2–2.8) 4 (4–4)	247 (200–300) 498 (500–500) 138 (138–138) 2 (2–3) 2.5 (2.5–3.5) 2.0 (1.1–2.8) 4 (4–4)	0.44 0.45 0.47 0.38 0.38 0.37 0.56
Pre-dialysis BP (mmHg), median (IQR) -Systolic blood pressure -Diastolic blood pressure -Mean arterial pressure	124 (111–140) 66 (58–74) 85 (77–94)	118 (104–132) 62 (57–70) 81 (74–87)	127 (115–142) 68 (59–77) 88 (80–96)	<0.001 <0.001 <0.001

Abbreviation: BP, blood pressure; BUN, blood urea nitrogen; DLC, double lumen catheter; HD, hemodialysis; IDH, intradialytic hypotension; IQR, interquartile range.

**Table 3:** Univariable and multivariable generalized estimating equations (GEE) analyses of risk factors associated with the development of IDH.

	Univariable OR			Multivariable OR		
	Odds Ratio	95% CI	*p*-Value	Odds Ratio	95% CI	*p* Value
Female	0.79	0.44–1.44	0.44			
Age > 65 years	1.41	0.78–2.57	0.25			
Body weight	1.00	0.97–1.02	0.78			
Height	1.01	0.97–1.04	0.69			
Baseline creatinine	1.00	0.93–1.08	0.95			
Residual urine < 100 mL/day	2.27	1.48–3.48	<0.001	1.78	1.17–2.71	0.007
Receiving vasopressor agent	3.43	2.02–5.83	<0.001	3.36	1.79–6.29	<0.001
Underlying disease -Type 2 diabetes -Hypertension -Ischemic heart disease -Atrial fibrillation	0.93 0.66 0.65 1.40	0.49–1.77 0.36–1.20 0.32–1.32 0.60–3.25	0.81 0.17 0.24 0.43			
Previous HD IDH	2.22	1.39–3.56	0.001	2.25	1.34–3.78	0.002
Pre-HD MAP ≥ 90 mmHg	Reference			Reference		
Pre-HD MAP 80–89 mmHg	2.36	1.41–3.95	0.001	2.43	1.37–4.28	0.002
Pre-HD MAP < 80 mmHg	3.31	2.04–5.39	<0.001	2.95	1.80–4.85	<0.001
Dialysis prescription -Dialysate sodium -Dialysate potassium 3 mEq/L -Dialysate calcium 2.5 mEq/L -Dialysate calcium 3.5 mEq/L -Blood flow rate -HD duration -Prescribed UF > 2 L/session	1.01 0.94 Reference 0.85 1.00 1.09 1.19	0.80–1.25 0.64–1.40 0.59–1.21 1.00–1.01 0.82–1.46 0.82–1.73	0.91 1.78 0.36 0.30 0.54 0.36			
Pre dialysis laboratories -BUN > 100 mg/dL -Creatinine > 5.5 mg/dL -Sodium < 135 mmol/L -Potassium < 4.0 mmol/L -Bicarbonate < 20 mmol/L -Calcium < 8.5 mg/dL -Albumin < 2.5 g/dL -Hematocrit < 25% -Glucose > 150 mg/dL	1.12 0.75 0.70 1.21 1.37 1.18 1.68 0.93 1.12	0.76–1.66 0.46–1.23 0.43–1.13 0.83–1.76 0.94–2.00 0.76–1.83 1.15–2.45 0.64–1.34 0.76–1.65	0.56 0.26 0.14 0.33 0.10 0.46 0.007 0.70 0.55	1.44	0.96–2.15	0.08

Abbreviation: BUN, blood urea nitrogen; HD, hemodialysis; IDH, intradialytic hypotension; MAP, mean arterial pressure; UF, ultrafiltration.

**Table 4:** Regression Coefficients of Variables Included in the IDH Risk Score.

Variable	Multivariable Coefficient	*p* Value	95% CI
Urine output < 100 mL/day	0.58	0.01	0.16 to 1.00
Receiving vasopressor agent	1.21	<0.001	0.58 to 1.84
Pre-HD MAP ≥ 90 mmHg	Reference		
Pre-HD MAP 80–89 mmHg	0.89	0.002	0.32 to 1.45
Pre-HD MAP < 80 mmHg	1.08	<0.001	0.59 to 1.58
Albumin < 2.5 g/dL	0.36	0.08	−0.04 to 0.77
Previous HD IDH	0.81	0.002	0.29 to 1.33

Abbreviation: HD, hemodialysis; IDH, intradialytic hypotension; MAP, mean arterial pressure.

**Table 5:** Prediction score for IDH in Acute kidney injury.

Variable	Point
Urine < 100 mL/day	2
Receiving vasopressor agent	3
Pre-HD MAP 80–89 mmHg	2
Pre-HD MAP < 80 mmHg	3
Albumin < 2.5 g/dL	1
Previous HD IDH	2

Abbreviation: HD, hemodialysis; IDH, intradialytic hypotension; MAP, mean arterial pressure.

## Data Availability

The data presented in this study are available on request from the corresponding author. The data are not publicly available due to privacy and ethical restrictions.
